# Laparoscopic repair of irreducible femoral hernia containing the fallopian tube alone: a case report

**DOI:** 10.1186/s40792-016-0185-y

**Published:** 2016-06-06

**Authors:** Nobutoshi Soeta, Takuro Saito, Tetsutaro Nemoto, Ikuro Oshibe, Mitsukazu Gotoh

**Affiliations:** Department of Surgery, Aizu Medical Center, Fukushima Medical University, 21-2 Maeda, Tanisawa, Kawahigashi, Aizuwakamatsu, Fukushima 969-3492 Japan; Department of Surgery, Fukushima Medical University, 1-Hikarigaoka, Fukushima, Fukushima 960-1295 Japan

**Keywords:** Fallopian tube, Femoral hernia, Laparoscopic hernia repair

## Abstract

**Background:**

We offer the first report of laparoscopic repair of an irreducible femoral hernia containing the fallopian tube alone.

**Case presentation:**

An 84-year-old woman presented with a 2-week history of a right groin mass with no abdominal symptoms. The mass was located below the inguinal ligament but showed no redness or tenderness. Abdominal computed tomography demonstrated a 4 × 3-cm cystic mass and enhanced cord-like structure in the right groin area. Hernia contents were considered potentially associated with the appendix, and right femoral hernia incarceration was diagnosed. We performed emergency surgery using a laparoscopic approach, revealing an irreducible femoral hernia containing the right fallopian tube, which was reduced laparoscopically. The reduced fallopian tube showed no ischemic changes, obviating the need for resection. No other abdominal organs such as the ovary, fimbriae of the fallopian tube, or appendix were incarcerated. We repaired the femoral hernia laparoscopically using a transabdominal preperitoneal approach with a mesh.

**Conclusions:**

A laparoscopic approach offers ready and accurate confirmation of incarcerated or irreducible organs, rapid recovery, and favorable cosmesis and should therefore be considered for the treatment of incarcerated or irreducible femoral hernia.

## Background

Femoral hernias account for approximately 2–8 % of all groin hernias [1]. Due to the small size of the defect in the femoral ring and the rigid ligamentous structures, incarceration is observed far more frequently with femoral hernia than with other abdominal hernias [2]. The small intestine and greater omentum are common contents, but the fallopian tube is rare. We report an unusual case of an 84-year-old woman with an irreducible femoral hernia containing the right fallopian tube, which was successfully reduced laparoscopically and repaired with a polypropylene mesh.

## Case presentation

An 84-year-old woman presented with a 2-week history of a mass in the right groin region and was referred to our hospital. She had no abdominal pain or tenderness, nausea, or anorexia and reported no pain in the right groin region. She had no specific medical or surgical history. Physical examination revealed a non-tender, irreducible right groin mass, located below the inguinal ligament.

**Fig. 1:**
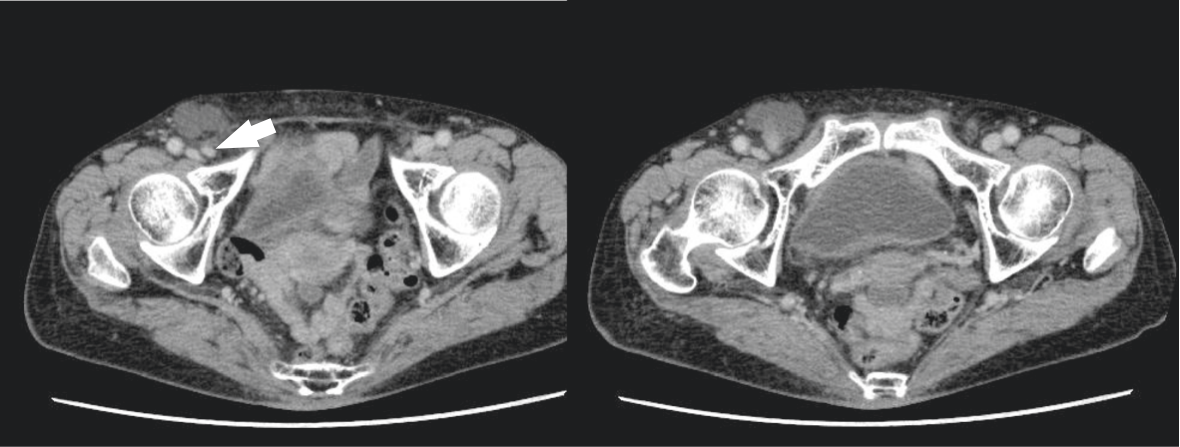
Preoperative findings of abdominal contrast-enhanced computed tomography. Abdominal contrast-enhanced computed tomography demonstrates a 4 × 3 cm cystic structure and enhanced cord-like structure (*arrowhead*) in the right groin region without the small bowel

Hematological and biochemical results were all within normal ranges, with no elevation of the white cell count or C-reactive protein level. Abdominal ultrasonography showed a solid and cystic mass in the right groin region continuous with the abdominal cavity. Abdominal contrast-enhanced computed tomography demonstrated a 4 × 3 cm cystic structure and enhanced cord-like structure in the right groin region with no small bowel (Fig. 1). Hernia contents were considered potentially associated with the appendix, leading to a presumptive diagnosis of femoral hernia incarceration. When we attempted manual reduction, she reported slight pain in the right groin and manual reduction was unsuccessful. We therefore decided to perform emergency surgery for the presumed incarcerated right femoral hernia.

**Fig. 2:**
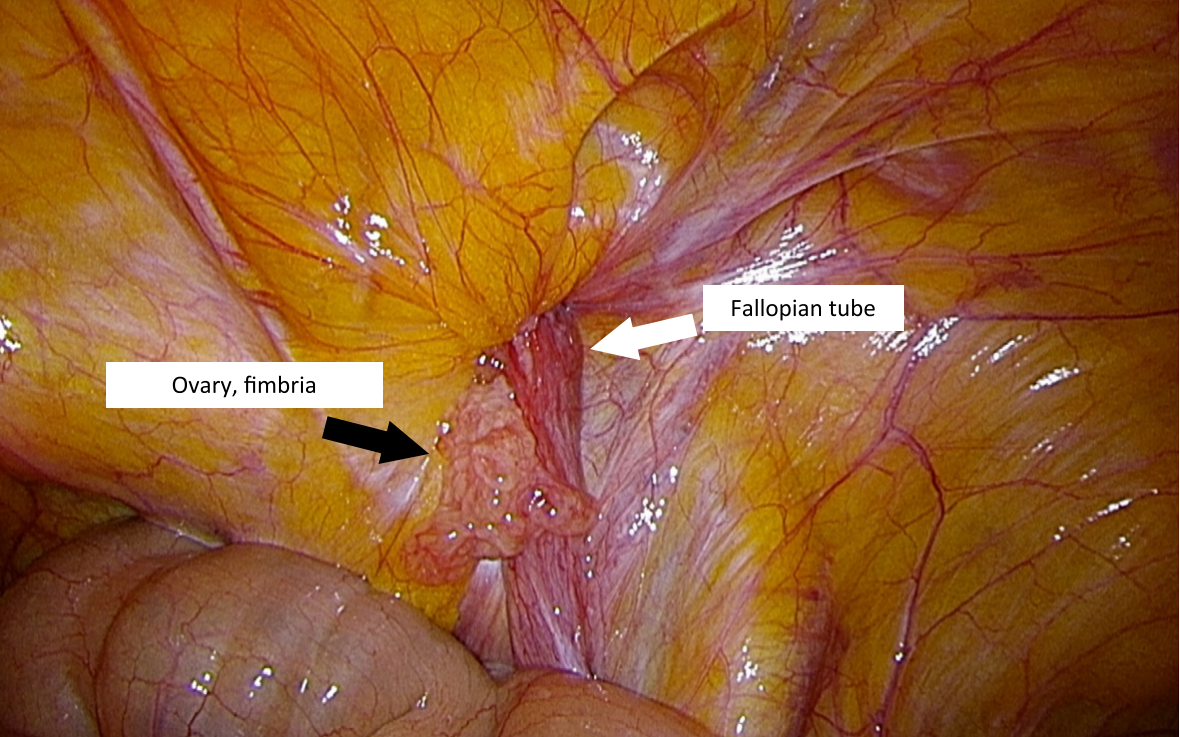
Operative findings with laparoscopic surgery before reduction. Contents of an irreducible right femoral hernia exposed using laparoscopic surgery: the fallopian tube (*white arrow*) is irreducible without any ovary or fimbriae of the fallopian tube (*black arrow*)

**Fig. 3:**
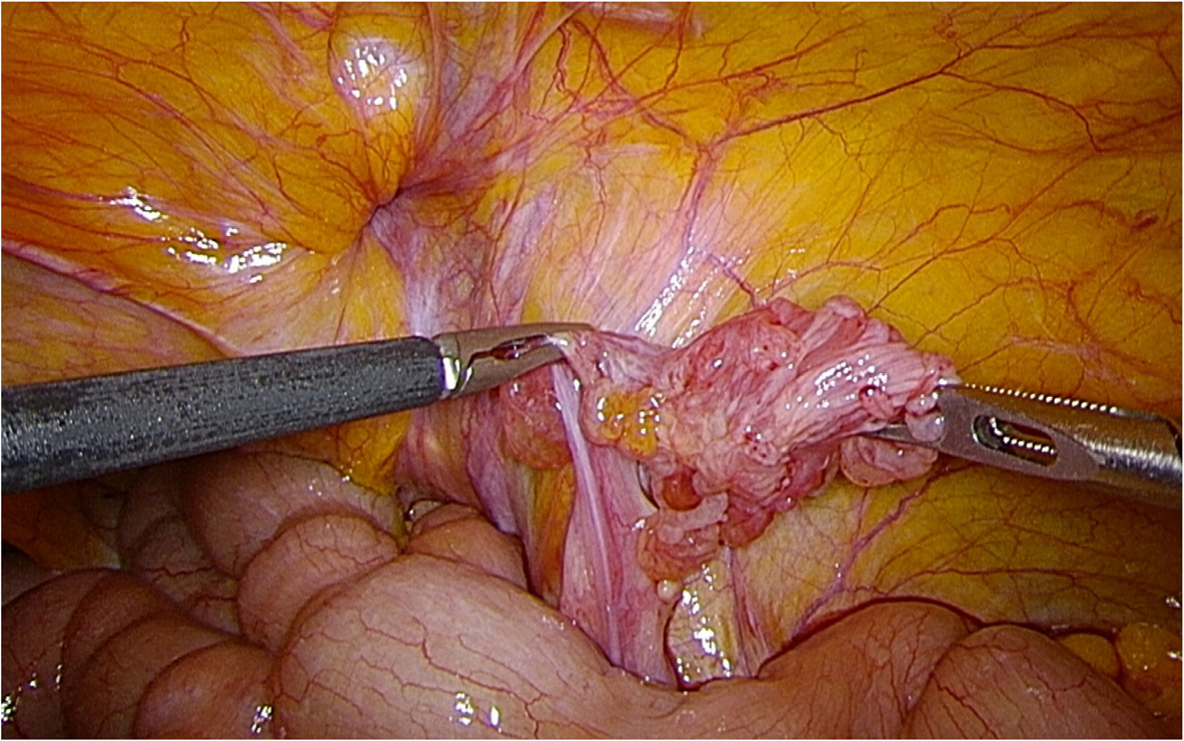
Operative findings with laparoscopic surgery after reduction. The irreducible fallopian tube shows no ischemic changes, and no anatomical abnormalities are evident in the fallopian tube, ovary, and fimbriae of the fallopian tube

The patient underwent laparoscopic repair of the femoral hernia using a transabdominal preperitoneal (TAPP) approach. Laparoscopic findings diagnosed a right irreducible femoral hernia containing the right fallopian tube (Fig. 2). No other abdominal organs such as the ovary, fimbriae of the fallopian tube, or appendix were incarcerated. The irreducible fallopian tube was successfully reduced laparoscopically and showed no ischemic change. The fallopian tube, ovary, and fimbriae showed no anatomical abnormalities (Fig. 3). The hernial orifice was approximately 1 cm in diameter. The hernial sac contained a small amount of serous fluid. We repaired the femoral hernia using a TAPP approach with a light-weight monofilament polypropylene mesh (3D Max™ Light mesh; Bard, Warwick, RI).

Postoperative recovery was uneventful, and the patient was discharged on postoperative day 3. No recurrence of femoral hernia was noted at the 3-month follow-up.

### Discussion

The incidence of femoral hernia is approximately 2–8 % in adults, comprising about 30 % of groin hernias [1]. This pathology is most commonly observed between the ages of 40 and 70 years and is very rare in younger ages. Femoral hernia is four to five times more common in women than in men, and a right-sided presentation is more common than left [1, 3]. Due to the small defect of the femoral ring and its rigid ligamentous structures, incarceration is observed far more frequently with femoral hernia than with other abdominal hernias [2]. Multiparity and the elevated intra-abdominal pressure that occurs in patients with constipation, obstructive lung disease, or pregnancy is implicated in the etiology [1, 3]. Femoral hernia is often associated with blood circulation disorder, which warrants emergency surgery [3]. In the present case, the femoral hernia was preoperatively considered to contain the appendix. When we attempted manual reduction, the patient reported slight pain in the right groin region and manual reduction was unsuccessful. We thus diagnosed an incarcerated femoral hernia, which necessitated emergency surgery. However, operative findings revealed the contents as the fallopian tube, which is not associated with blood circulation disorder. As a result, the femoral hernia was not “incarcerated” but rather “irreducible,” and emergency surgery was unnecessary in the present case.

The contents of an incarcerated femoral hernia are generally the small intestine or greater omentum. Rare contents of incarcerated or irreducible femoral hernias reported in the literature include the appendix, bladder, Meckel’s diverticulum, ectopic testis, stomach, and gynecological organs [4]. In terms of incarcerated femoral hernia containing gynecological organs such as the ovary, uterus, or fallopian tube, 11 cases have been reported in adults (Table 1) [5–14]. Of these cases, the ovary alone was present in two cases and the fallopian tube alone in six cases. Three reports described the necessity for resection of the incarcerated fallopian tube and ovary.

**Table 1:** Clinical characteristics of adult patients with incarcerated or irreducible femoral hernia containing gynecological organs

No.	Author	Year	Sex	Age (years)	Presenting symptoms	Affected side	Contents of hernia	Surgical approach	Resection	Use of a mesh	Outcome
1	Maylard AE [5]	1892	F	75	Irreducible inguinal swelling, pain	Left	Ovary	OS	+	−	Uneventful
2	Parkes CH [6]	1910	F	33	Irreducible inguinal swelling, pain	Right	Fallopian tube	OS	+	−	Unknown
3	Devane JF [7]	1916	F	Unknown	Lower abdominal pain	Right	Fallopian tube	OS	+	−	Uneventful
4	Keasling JE [8]	1959	F	43	Irreducible inguinal swelling, pain	Right	Ovary	OS	−	−	Uneventful
5	Atmatzidis S [9]	2010	F	20	Irreducible inguinal swelling, pain	Right	Fallopian tube	OS	−	+	Uneventful
6	Coyle D [10]	2011	F	54	Irreducible inguinal swelling, pain	Left	Ovary and fallopian tube	OS	−	−	Uneventful
7	Alzaraa A [11]	2011	F	39	Irreducible inguinal swelling, pain	Right	Fallopian tube	OS	−	−	Uneventful
8	Lopez C [12]	2011	F	47	Irreducible inguinal swelling, pain	Right	Fallopian tube	OS	−	+	Uneventful
9	Ay A [13]	2012	F	76	Irreducible inguinal swelling, pain	Right	Ileum, uterus, and both ovaries	OS	−	+	Uneventful
10	Ambedkar V [14]	2016	F	28	Irreducible inguinal swelling, pain	Left	Uterus, fallopian tube, and ovary	OS	−	−	Uneventful
11	Present case	2016	F	84	Irreducible inguinal swelling	Right	Fallopian tube	LS	−	+	Uneventful

*F* female, *OS* open surgery, *LS* laparoscopic surgery

The anatomical location of the ovaries, uterus, and fallopian tube at a level below the femoral ring makes herniation of these structures unusual, particularly in adults [11]. Acquired weakness in the pelvic wall with multiparity and/or elevated intra-abdominal pressure is thought to play an etiological role in the development of femoral hernias in adults [1]. In the present case, the contents of the femoral hernia were laparoscopically confirmed as the fallopian tube alone, showing no abnormalities of the uterus or uterine appendages.

The first recorded case of femoral hernia containing the fallopian tube alone, without the ovary, was described by Parkes in 1910 [6]. Since then, incarcerated femoral hernia containing the fallopian tube alone in adults has remained extremely rare, with only six cases reported (Table 1) [6, 7, 9, 11, 12]. These cases all had right-sided presentations. To the best of our knowledge, two case reports have described the necessity for resection of the incarcerated fallopian tube. Our report offers the first description of laparoscopic repair for an irreducible femoral hernia containing the fallopian tube alone.

Three different surgical approaches (femoral, inguinal, and laparoscopic) can be considered to repair femoral hernia, and all surgical modalities are in standard use by surgeons today. In our patient, we used a light-weight monofilament, polypropylene mesh herniorrhaphy technique with a laparoscopic TAPP approach.

The laparoscopic approach facilitates confirmation of the incarcerated or irreducible organs and any ischemic changes and accurately prevents the omission of any coexisting lesion. The laparoscopic approach may be suitable for patients in terms of fast recovery, easy and accurate confirmation of incarcerated or irreducible organs, and cosmetic outcomes.

## Conclusions

We have reported a case of laparoscopic repair of a femoral hernia containing a rare irreducible fallopian tube. In this case, a laparoscopic approach was successfully applied and offered the advantages of easy and accurate confirmation of incarcerated or irreducible organs, fast recovery, and favorable cosmetic outcomes. The laparoscopic approach should thus be considered for the treatment of incarcerated or irreducible femoral hernia.

## Consent

Written informed consent was obtained from the patient for publication of this case report and the accompanying images. A copy of the written consent is available for review by the Editor-in-Chief of this journal.

## Abbreviation

TAPP, transabdominal preperitoneal
